# Flexible Phase Change Materials with High Energy Storage Density Based on Porous Carbon Fibers

**DOI:** 10.3390/polym16243547

**Published:** 2024-12-19

**Authors:** Xiangqin Peng, Lei Chen, Bohong Li, Zhe Tang, Yifan Jia, Zhenyu Jason Zhang, Qianqian Yu, LinGe Wang

**Affiliations:** 1 Guangdong Provincial Key Laboratory of Functional and Intelligent Hybrid Materials and Devices, Guangdong Basic Research Center of Excellence for Energy and Information Polymer Materials, State Key Laboratory of Luminescent Materials and Devices, South China Advanced Institute for Soft Matter Science and Technology, School of Emergent Soft Matter, South China University of Technology, Guangzhou 510640, China; 202120162318@mail.scut.edu.cn (X.P.); lchan_95@163.com (L.C.); chinahong4211@163.com (B.L.); 202310192840@mail.scut.edu.cn (Z.T.); kyvjyf@163.com (Y.J.); 2 School of Chemical Engineering, University of Birmingham, Birmingham B15 2TT, UK

**Keywords:** flexible phase change materials, high energy storage density, thermal energy storage, porous carbon fibers

## Abstract

Phase change fibers (PCFs) can effectively store and release heat, improve energy efficiency, and provide a basis for a wide range of energy applications. Improving energy storage density and preserving flexibility are the primary issues in the efficient manufacture and application development of PCFs. Herein, we have successfully fabricated a suite of flexible PCFs with high energy storage density, which use hollow carbon fibers (HCFs) encapsulated phase change materials (PCMs) to provide efficient heat storage and release, thereby enhancing energy efficiency and underpinning a broad range of energy applications. The flexible HCF/LA PCFs with high energy density were made by impregnating a small molecule LA solution, whereas the precursor of the PAN/ZIF-67 composite fibers was created by electrospinning. These PCFs have a high loading capacity for lauric acid (LA), demonstrating a 92% load percentage and a 153 J g^−1^ phase change enthalpy value. The effects of doping quantity (ZIF-67), fiber orientation, pre-oxidation treatment, and particle size on the morphological and structural characteristics of HCFs, as well as the impact of HCFs’ pore structure on PCM encapsulation, were investigated. It was found that the oriented fiber structure serves to reduce the likelihood of fracture and breakage of precursor fibers after carbonization, whilst the gradient pre-oxidation can maintain the original fiber morphology of the fibers after carbonization. These findings establish a solid theoretical foundation for the design and production of high-performance flexible porous carbon nanofiber wiping phase change composites.

## 1. Introduction

Over the past few decades, both the increasing demand for energy due to technological improvements and population increase, and the continual emission of greenhouse gases from conventional fossil fuels urge research and innovation in securing green and renewable energy sources, with an improved energy efficiency [1]. Among them, the deployment of phase change materials (PCMs) for latent heat storage presents an effective method for optimizing thermal energy use, addressing the temporal and geographical discrepancies between thermal energy supply and demand, thereby providing a promising solution for mitigating an increasing global energy demand and encouraging a carbon neutral future.

PCMs are excellent latent heat energy storage materials that are gaining popularity due to their high energy storage density within a narrow temperature range [2,3,4,5]. Compared to other storage technologies such as sensible heat storage and chemical reaction storage, phase change storage has the benefits of high energy density, small equipment size, simple materials, and a constant temperature during the phase change process, as well as a wide range of applications [6,7]. Organic solid–liquid PCMs, such as wax and fatty acids, offer high phase change enthalpy, small volumetric expansion, no overcooling or phase separation, non-toxicity, and corrosion resistance, and are widely used [8,9,10]. However, they are susceptible to leakage during phase transition. As a result, research on organic phase change energy storage materials has been primarily focused on addressing the leakage issue [11,12,13], including innovative packaging techniques to minimize phase change materials. In comparison to macro-scale packaging, nano-scale packaging technology has been reported to prepare stable phase change composites due to its advantages of a large specific surface area, strong surface interaction, diverse surface functions, controlled volume expansion, and high heat transfer rate [14]. In addition, the flexibility of PCMs is critical to their performance in a variety of engineering applications [15]. Flexible PCMs can withstand deformation such as bending and torsion, which allows them to better fit with the target object, thus providing better performance in terms of thermal management. This adaptability is particularly important in applications such as thermal management of electronic devices, thermal therapy, and flexible sensors [16,17,18,19,20,21,22].

When it comes to constructing flexible multiscale porous fiber scaffolds, electrospinning has demonstrated an unparalleled technological advantage [23,24,25]. Different morphologies of micro- and nanofibers can be produced by varying the spinning process [26,27]. Using a sacrificial template approach, the pore-causing agent is blended with the fiber to create the inner pore, and the sacrificial component pore-causing agent is removed through post-treatment subsequently. It is possible to construct flexible porous carbon fiber with high porosity, a multiscale porous structure, and exceptional thermal conductivity by calcining an electrospinning precursor at a high temperature [16,18,28,29]. Such fiber has a great potential for encapsulating PCMs. Polyacrylonitrile (PAN) has exceptional chemical stability, oxidation resistance, and solvent resistance because the nitrogen atom on the cyanide group forms a hydrogen bond with the hydrogen atom on the adjacent chain and the side cyano-group on the macromolecular chain has a strong polarity that gives it a strong intermolecular force [30,31]. ZIF-67 is a metal-organic frameworks (MOFs) material, which is a porous crystal material formed by connecting metal ions (usually transition metal ions) and organic ligands via coordination bonds. ZIF-67 has a very high specific surface area, which gives it a wide range of potential applications in gas storage, separation, and catalysis [32,33]. At the same time, a rich multiscale porous structure can be produced, and the skeleton structure of the precursor can be preserved after carbonization due to the high number of carbon atoms in its organic ligand molecular structure. It can be used to manufacture porous carbon compounds as a precursor and pore-inducing agent [34,35,36].

**Figure 1:**
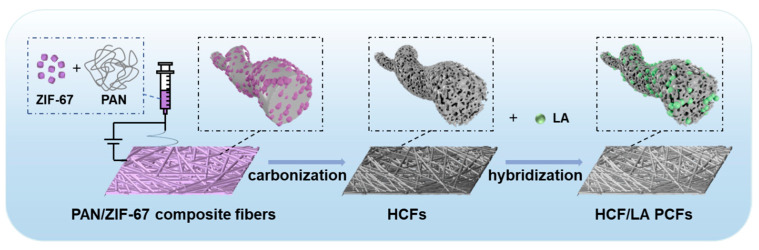
Schematic diagram of the preparation process of PAN/ZIF-67 composite fibers, HCFs, and HCF/LA PCFs.

In this study, we successfully used the co-precipitation method to prepare ZIF-67 nanoparticles of different sizes, which acted as templates for pore formation and carbonization precursors. Electrospinning was subsequently used to develop the precursor of the PAN/ZIF-67 composite fibers. Hollow carbon fibers (HCFs) with multiscale pore structure were created by pre-oxidizing the precursor using a gradient and calcining it at high temperatures. To achieve high efficiency load encapsulation of PCM via a porous fiber membrane, a small molecule material solution was impregnated to generate the HCF/LA phase change fibers (PCFs) (Figure 1). Additionally, the impacts of ZIF-67 template doping quantity, particle size, high-temperature calcination, fiber orientation, and pre-oxidation treatment on the morphology and pore structure of HCF were examined, as was the impact of HCFs’ porous structure on the packing effect of PCM. These findings provide significant insight for the design and preparation of flexible phase change composite materials with high energy storage density.

## 2. Materials and Methods

### 2.1. Materials

PAN powder (P60C, *M*_w_ = 150,000) was purchased from Shanghai Sigma Aldrich Trading Co., Ltd. (Shanghai, China). *N*. *N*-dimethylformamide (DMF) was purchased from Shanghai Titan Chemical Co., Ltd. (Shanghai, China). Cobalt nitrate hexahydrate (Co(NO_3_)_2_·6H_2_O) and 2-methylimidazole (2-MI) were purchased from Shanghai Adamas Reagent Factory (Shanghai, China). Methanol was purchased from Guangzhou Chemical Reagent Factory (Guangzhou, China). LA was purchased from Shanghai Ronen Chemical Reagent Factory (Shanghai, China). All chemicals were used without further purification.

### 2.2. Preparation of ZIF-67 and PAN/ZIF-67 Composite Fibers

The ZIF-67 was prepared using a co-precipitation process [37] by adding 18 g of 2-MI to 40, 80, and 100 mL of deionized water for ultrasonic dissolution after equally dissolving 1.2 g of Co(NO_3_)_2_·6H_2_O in 10, 20, and 25 mL of deionized water, respectively. The two solutions were separately combined and mixed with a magnetic stirrer set to 800 rpm for 2 h at 25 °C. ZIF-67 precipitation was produced and centrifugally cleaned. Various sizes of ZIF-67 powder were obtained and freeze-dried.

Electrospinning was used to produce the precursory PAN/ZIF-67 composite fibers. To prepare a spinning solution with a PAN concentration of 10%, PAN powder was fully dissolved in DMF. To prepare a PAN/ZIF-67 composite spinning solution, ZIF-67 powder was added and thoroughly dissolved. A custom-build uniaxial electrospinning machine was used to manufacture the composite fibers (TL-Pro, Shenzhen Tongli Micro Nano Technology Co., Ltd. (Shanghai, China)) [38]. A drum collects fibers during the electrospinning process, which takes place in a high-voltage electrospinning machine at 25 °C and 50% relative humidity. The receiving distance is 20 cm, the receiving drum speed is 2000 rpm (or 100 rpm), the spinning voltage is 15 kV, and the spinning solution injection speed is 1 mL per hour. The obtained PAN/ZIF-67 composite fiber membrane was dried for 12 h in a 60 °C oven.

### 2.3. Preparation of HCFs and HCF/LA PCFs

HCFs were fabricated using gradient pre-oxidation and high-temperature carbonization. Pre-oxidized composite fibers were created by subjecting the precursor PAN/ZIF-67 composite fibers to constant temperature oxidation at 180 °C, 200 °C, 220 °C, and 240 °C for 0.5 h and 250 °C for 1 h in an air atmosphere. To produce porous carbon fibers, pre-oxidized composite fibers were carbonized at a rate of 5 °C min^−1^ to 800 °C in an argon atmosphere for 3 h.

To prepare HCF/LA PCFs, 200 mg of HCFs was submerged in a methanol solution containing 200 mg/mL of LA for a duration of 24 h. Methanol and deionized water were then used to clean it. The phase change composite fibers HCF/LA were created after 12 h of drying at 37 °C.

### 2.4. Characterization

Scanning electron microscopy (SEM) and transmission electron microscopy (TEM) were utilized to investigate the geometry and structure of ZIF-67 and other fibers. ZIF-67 powder (or fiber) was placed on a sample stage for actual sputtering gold plating and examined under an SEM. ZIF-67 powder (or fiber) was applied on a carbon film copper mesh for TEM examination. The size distribution of ZIF-67 was calculated using ImageJ 1.48v software. X-ray diffraction (XRD) was utilized to determine the crystal structure of ZIF-67 and precursor PAN/ZIF-67 composite fibers. The scanning range was 2θ = 5~50°, with a scanning time of 25 s. HCFs’ pore characteristics were examined using Brunner–Emmet–Teller (BET) measurements (Belsorp-Max, MicrotracBEL, Inc., Osaka, Japan). The specific surface area was measured using the nitrogen absorption–desorption method. The size distribution of pores was estimated using the Barrett–Joerner–Halenda (BJH) model.

Differential scanning calorimetry (DSC) was used to examine the PCFs melting and crystallization processes as well as the enthalpy of phase change. With a program that alternates between 20 °C and 80 °C and again between 80 °C and 20 °C, the heating rate was 10 °C min^−1^. The testing atmosphere was nitrogen, and the temperature was maintained at a fixed level for two minutes following each temperature rise and fall. The composition of PAN/ZIF-67 composite fibers and the loading of PCMs into phase change composite fibers were examined using a thermo-gravimetric analyzer (TGA). The test was conducted in a dish type thermogravimetric analyzer, with a temperature range of 30–800 °C, a heating rate of 10 °C/min, and a testing atmosphere of N_2_.

## 3. Results and Discussion

### 3.1. Preparation and Characterization of PAN/ZIF-67 Composite Fibers

**Figure 2:**
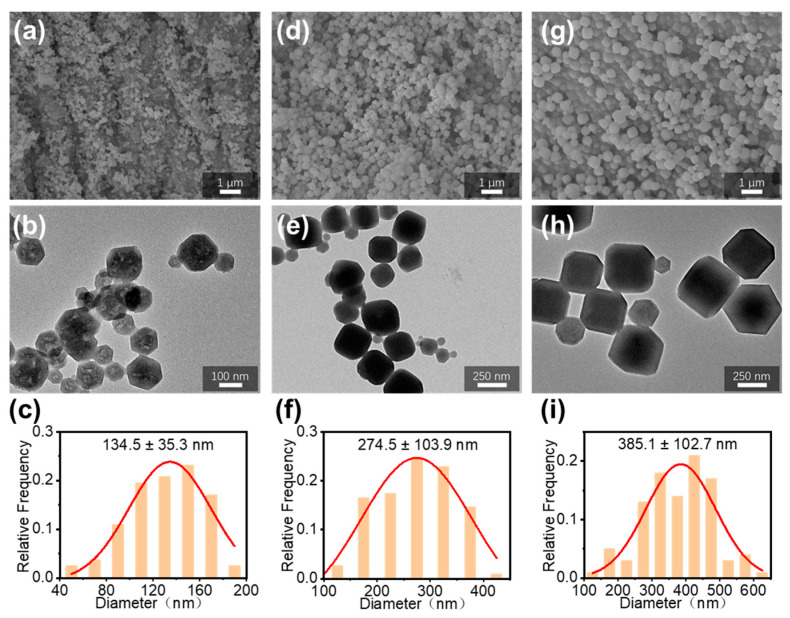
(**a**,**d**,**g**) SEM and (**b**,**e**,**h**) TEM images of three different particle sizes of ZIF-67, and the corresponding particle size statistics (**c**,**f**,**i**) of the SEM images.

The pore-forming agent, ZIF-67 nanoparticles, was prepared by co-precipitation of Co(NO_3_)_2_·6H_2_O and 2-MI in water. Three different particle sizes of ZIF-67 were prepared by adjusting the concentration and ratio of reactants. Since the diameter of PAN nanofibers is about 800 nm, the particle size of added ZIF67 nanoparticles should be smaller than the diameter of pan fibers in order to better maintain the morphology and structure of composite fibers. Therefore, ZIF67 of about 150 nm, 300 nm, and 400 nm were synthesized for comparison. The morphology and structure of ZIF-67 were characterized by SEM and TEM, as shown in Figure 2. Three different sizes of ZIF-67, in the form of granular rhombic dodecahedral structures, alongside their hexagonal cross sections, can be observed in the TEM images. The molar ratios of three different particle sizes of ZIF-67 were Co^2+^/2-MI/water: 1/53/675 (Figure 2a–c), 1/53/1350 (Figure 2d–f), and 1/53/1685 (Figure 2g–i), respectively. Based on the SEM images, the averaged particle sizes of ZIF-67, determined by Image J, were around 134.5 nm, 274.5 nm, and 385.1 nm, respectively. It shows that the ZIF-67 particle size increases progressively when the dosage of solvent water is increased. The particle size distribution of the three groups of ZIF-67 has a certain width, so they are referred to as 150 nm ZIF-67, 300 nm ZIF-67, and 400 nm ZIF-67 based on their approximate range of particle size.

**Figure 3:**
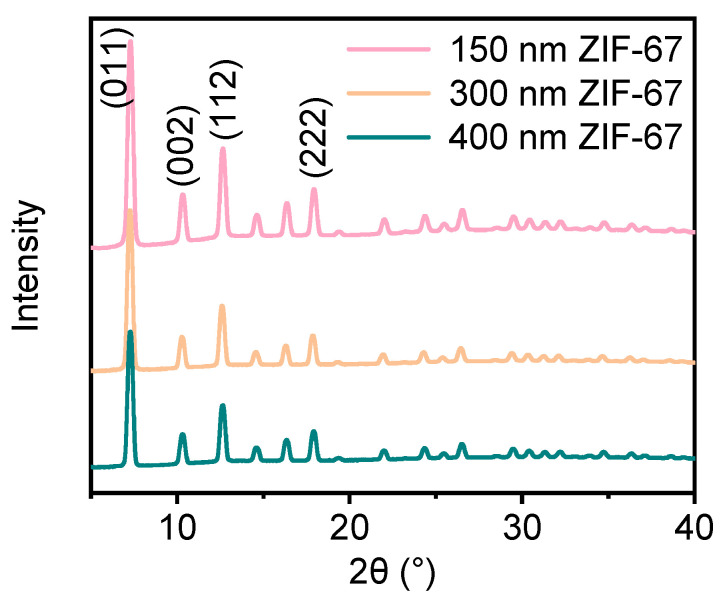
XRD diffraction patterns of three different particle sizes of ZIF-67.

The crystal structures of three groups of ZIF-67 were characterized by XRD, as shown in Figure 3. The three groups of ZIF-67 with different sizes present the same diffraction peaks, with four prominent characteristic peaks appearing at 2θ = 7.3°, 10.3°, 12.6°, and 17.9°, which correspond to the (011), (002), (112), and (222) crystal planes. The observation is consistent with those reported in the literature [39] and without other impurity peaks, confirming that the synthesized ZIF-67 material is of high purity.

**Figure 4:**
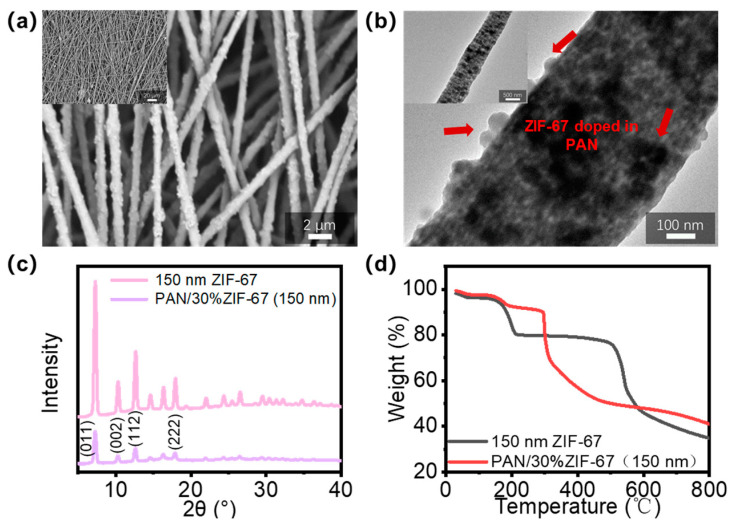
(**a**) SEM and (**b**) TEM results of PAN/30% ZIF-67 (150 nm) composite fibers; (**c**) XRD diffraction pattern of PAN/30% ZIF-67 (150 nm) composite fibers; (**d**) Thermogravimetric curves of ZIF-67 nanoparticles and PAN/30% ZIF-67 (150 nm) composite fibers.

Electrospinning was then used to fabricate PAN/ZIF-67 composite fibers. Composite fibers doped with 30 weight percent ZIF-67 of PAN mass were named as PAN/30% ZIF-67 (150 nm), while PAN/ZIF-67 composite fibers with varying doping contents and particle sizes were assigned in the same manner. This is based on the various doping contents and particle sizes of ZIF-67. SEM and TEM were used to investigate the morphology and structure of the composite fibers. Figure 4a,b presents the fiber surface as rough and granular, as well as the distribution of ZIF-67 on the fiber surface in the SEM images (red arrow). The fibers have a diameter of approximately 800 nm and are relatively uniform. TEM images reveal that many black particles with enhanced contrast are consistently dispersed inside the fiber, indicating that ZIF-67 nanoparticles have been doped into it. The findings of both electron microscopy experiments indicate that ZIF-67 nanoparticles had been successfully doped into the PAN fiber matrix and dispersed uniformly, providing an excellent basis for the subsequent high-temperature carbonization of HCFs.

XRD analysis of PAN/30% ZIF-67 (150 nm) composite fibers confirmed ZIF-67 doping in PAN fibers. The composite fibers’ diffraction curve (Figure 4c) reveals typical peaks corresponding to ZIF-67, demonstrating the successful composite of ZIF-67 in PAN fibers, while the original crystal structure remains intact. The thermal degradation of ZIF-67 nanoparticles and PAN/ZIF-67 composite fibers was investigated using TGA, as shown in Figure 4d. The weight loss curve for PAN/30% ZIF-67 (150 nm) composite fibers is separated into three segments. Weight loss at 150–200 °C is caused by ZIF-67 breakdown, while rapid weight loss between 300–500 °C is mostly caused by PAN cyclization and dehydrogenation. PAN thermal decomposition stabilizes at temperatures above 500 °C, resulting in carbonaceous residues. The TGA test findings confirm the effective doping of ZIF-67 nanoparticles in PAN fibers and provide recommendations for subsequent carbonization temperatures.

### 3.2. Preparation and Characterization of HCFs

A sacrificial template approach was used to prepare PAN-based HCFs, whereby the precursor PAN/ZIF-67 composite fiber was calcined at high temperatures to produce HCFs with ZIF-67 nanoparticles as the pore-forming agent, of which the parameters influencing its morphology and structure were investigated. PAN/ZIF-67 composite fiber must be pre-oxidized before being calcined at high temperatures. The oxidation reaction introduces hydroxyl and carbonyl groups into fiber molecules, and hydrogen bonds are formed between and within the molecules, gradually transforming PAN fibril into pre-oxidized fiber with trapezoidal structure, which improves thermal stability and prevents fibril melting during carbonization. Thus, the fiber structure is preserved during the subsequent high-temperature calcination [36].

**Figure 5:**
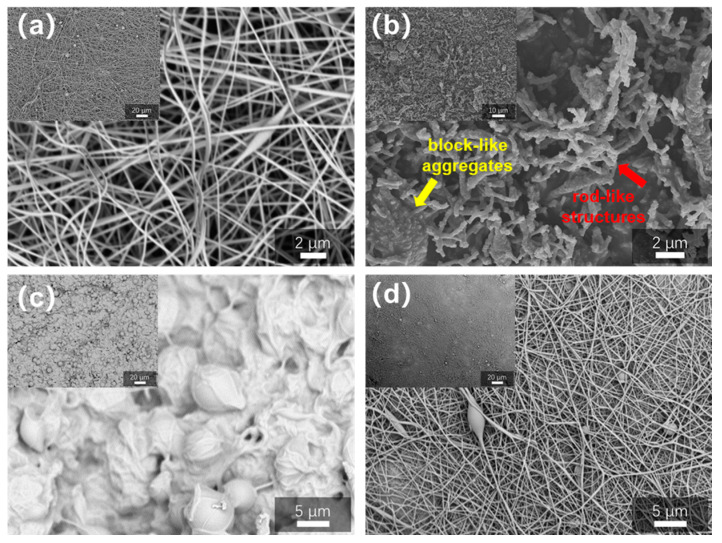
SEM image of (**a**) PAN fibers precursor, (**b**) Directly carbonize at 800 °C for 3 h, (**c**) Directly pre-oxidize at 250 °C for 3 h and then carbonize at 800 °C for 3 h, (**d**) gradient pre-oxidation followed by carbonization at 800 °C for 3 h.

Firstly, appropriate pre-oxidation process parameters were investigated using pure PAN fibers. The choice of pre-oxidation temperature is consistent with the process in the conventional preparation of carbon fiber, and the temperature used in the relevant literature is 250 °C [40,41], and 800 °C is the characteristic temperature of high temperature carbonization reaction. The appearance and structure of the final carbonized product were examined by SEM after PAN fibers treated with various pre-oxidation treatments were carbonized for three hours at 800 °C, as illustrated in Figure 5. The PAN fiber precursor made by electrospinning is presented in Figure 5a, in which a randomly distributed fiber structure and a small quantity of beads were observed. The product of immediately carbonizing PAN precursor fibers at 800 °C without pre-oxidation is displayed in Figure 5b. It is evident that the fibers had all disintegrated into small rod-like structures (red arrow), which is distinctively different from the initial continuous fiber structure. Certain densely populated regions melted and solidified as block-like aggregates (yellow arrow). This is because, during high-temperature pyrolysis, PAN precursor fibers that have not been pre-oxidized are more likely to break their chains and change into resin carbon instead of fibrous carbon with a certain strength. Figure 5c shows the product generated by directly heating the PAN precursor to 250 °C for pre-oxidation for 3 h and then carbonizing it at 800 °C. It is noticeable that the physical appearance and structure of the fibers had diminished completely. This is very likely because the direct heating rate is too high, and the PAN precursor has not yet changed into pre-oxidized fibers with a trapezoidal shape in time, causing the fiber structure to melt and be destroyed. Carbon fibers were generated by gradient pre-oxidation of PAN precursor and carbonization at 800 °C, as shown in Figure 5d. Gradient pre-oxidation involves 0.5 h of constant temperature oxidation at 180 °C, 200 °C, 220 °C, and 240 °C, followed by one hour of constant temperature oxidation at 250 °C. Carbon fibers generated using gradient pre-oxidation can effectively preserve the original fiber structure, preventing fracture, breakage, and melting. As a result, in the next preparation of porous carbon fibers in this chapter, this gradient pre-oxidation procedure was employed to pretreat PAN/ZIF-67 composite fibers.

**Figure 6:**
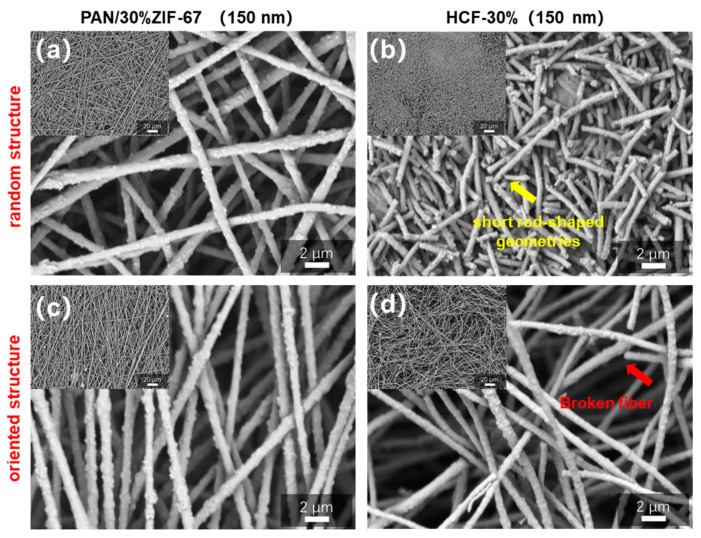
SEM images of (**a**) random PAN/30% ZIF-67 (150 nm) fibers received through a 100 rpm drum and (**b**) porous carbon fibers prepared by high-temperature calcination, SEM images of (**c**) oriented PAN/30% ZIF-67 (150 nm) fibers received through a 2000 rpm drum and (**d**) porous carbon fibers prepared by high-temperature calcination.

The orientation structure of precursor fibers has a substantial impact on the morphology, structure, and physical properties of carbonized fibers [42]. Two sets of PAN/30% ZIF-67 (150 nm) composite fibers with random structure (*n* = 100 rpm) and oriented structure (*n* = 2000 rpm) were prepared by changing the rotational speed of the electrospinning receiving device. Figure 6a depicts the SEM image of randomly oriented PAN/30% ZIF-67 (150 nm) composite fibers with a chaotic and staggered orientation. Figure 6b depicts HCFs made from random PAN/30% ZIF-67 (150 nm) fibers that fracture and break into short rod-shaped geometries (yellow arrow), failing to retain the macroscopic fiber morphology. Figure 6c exhibits PAN/30% ZIF-67 (150 nm) composite fibers with a highly orientated structure. Figure 6d shows the porous carbon fiber made from orientated PAN/30% ZIF-67 (150 nm) fibers. After pre-oxidation and carbonization, it appears that the fibers reduce in size, bend, and curl, but preserve a rather full fiber shape and orientation, with no large-scale fracture or breakage (red arrow). This is because oriented fibers can contract and release stress in the axial direction, preventing fiber fracture and keeping a somewhat full fiber shape following carbonization. The foregoing results show that precursor PAN/30% ZIF-67 (150 nm) composite fibers with an orientated structure can effectively sustain the intact fiber structure after carbonization.

**Figure 7:**
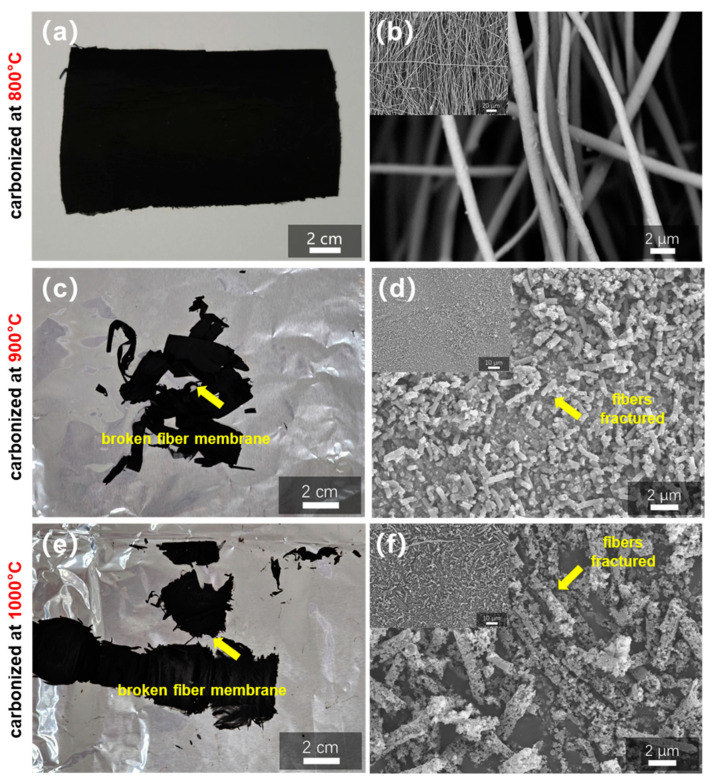
(**a**) Photograph and (**b**) SEM images of PAN/30% ZIF-67 (150 nm) fibers with gradient pre-oxidation carbonized at 800 °C, (**c**,**d**) carbonized at 900 °C, and (**e**,**f**) carbonized at 1000 °C.

The carbonization temperature is a crucial factor in the preparation of porous carbon fibers. Higher carbonization temperatures can result in larger specific surface areas, but they can also increase the carbon content and brittleness of the final fibers, making them more susceptible to fracture [40]. As a result, it is critical to optimize the carbonization temperature. Carbonization temperatures for orientated PAN/30% ZIF-67 (150 nm) fibers with gradient pre-oxidation were 800 °C, 900 °C, and 1000 °C. The products’ shape and structure were examined using SEM. In contrast to fibers carbonized at 900 °C and 1000 °C, which fractured considerably and produced a broken fiber membrane (yellow arrow), Figure 7 shows that only fibers carbonized at 800 °C retained their original morphology and were able to obtain a complete fiber membrane. It can be concluded that 800 °C is the optimal carbonization temperature for the HCFs used in the present work.

Another important factor that affects the ultimate synthesis of HCFs’ porous structures is the dosage and size of ZIF-67. PAN/ZIF-67 composite fibers with different ZIF-67 doping concentrations and particle sizes were made by electrospinning, while HCFs were made by gradient pre-oxidation and carbonization at 800 °C. To investigate the impact of ZIF-67 doping content and particle size on the porous structure of the produced HCFs, SEM and TEM investigations were conducted on the morphology and structure of the original PAN/ZIF-67 composite fibers as well as the finished HCFs.

**Figure 8:**
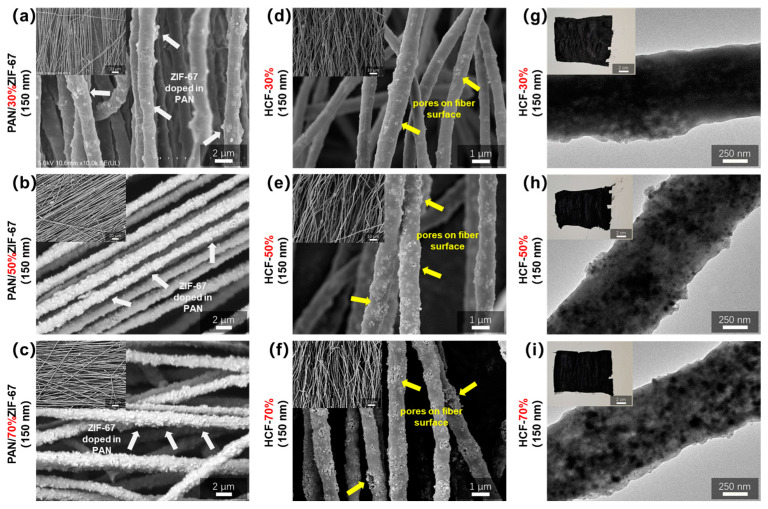
SEM images of PAN/ZIF-67 composite fibers with three different 150 nm ZIF-67 doping contents: (**a**) 30%, (**b**) 50%, (**c**) 70%; (**d**–**f**) SEM and (**g**–**i**) TEM images and photographs of HCF prepared from three groups of PAN/150 nm ZIF-67 composite fibers with different doping contents.

Figure 8 displays SEM and TEM images of PAN/ZIF-67 composite fibers with three distinct ZIF-67 and HCF doping concentrations (white arrow). As the ZIF-67 doping level rises, the number of particles on the PAN/ZIF-67 composite fiber surface increases, but the distribution is very uniform and there is no discernible agglomeration. As the ZIF-67 doping level grows, the pore distribution is rather uniform, as seen by its HCF, which shows that the particles inside the original composite fiber turn into pores (yellow arrow). When the doping quantities surpass 70% of PAN, the pore groups in the HCF derived from ZIF-67 aggregate and form a three-dimensional interconnected network structure.

**Figure 9:**
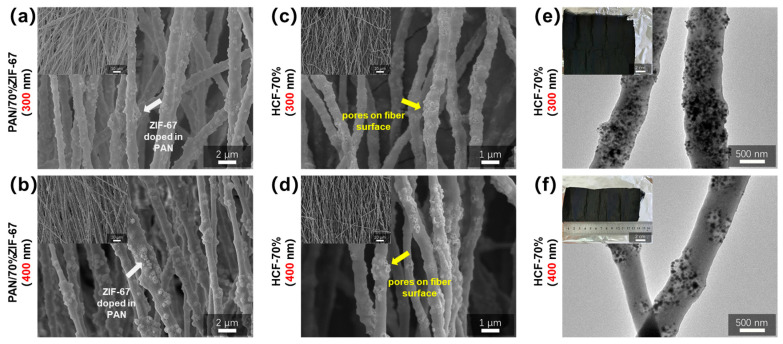
SEM images of three groups of PAN/ZIF-67 composite fibers doped with ZIF-67 of different sizes: (**a**) 300 nm ZIF-67, (**b**) 400 nm ZIF-67, (**c**,**d**) SEM, and (**e**,**f**) TEM images and photographs of HCF prepared from three groups of PAN/ZIF-67 composite fibers with different doping sizes.

ZIF-67 with a particle size of around 150 nm exhibits good dispersion in composite fibers, as shown in Figure 8c. Figure 9 shows SEM and TEM images and photographs of PAN/ZIF-67 composite fibers doped with 300 nm and 400 nm diameters of ZIF-67 and HCF synthesized from them. The dispersion of nanoparticles in PAN/ZIF-67 composite fibers becomes irregular as ZIF-67 particle size increases (white arrow). Large beads emerge from the fiber surface as a result of significant agglomeration that occurs when the particle size surpasses 400 nm. HCF composed of three different kinds of composite fibers exhibits the same pattern. HCF-70% (150 nm) has an evenly distributed three-dimensional interconnected pore structure, whereas HCF-70% (300 nm) and HCF-70% (400 nm) have an uneven internal pore distribution (yellow arrow) and an evident aggregation phenomenon. The aforementioned results show that increasing the amount of ZIF-67 doping increases the prepared HCF’s porous structure and porosity. However, as the particle size of ZIF-67 doping rises, the pore distribution of HCF becomes uneven, resulting in aggregation. HCF-70% (150 nm), made from PAN/70% ZIF-67 (150 nm) composite fibers, has the most abundant three-dimensional interconnected pore structure, making it an excellent structural foundation for loaded phase transition materials or other functional materials.

**Figure 10:**
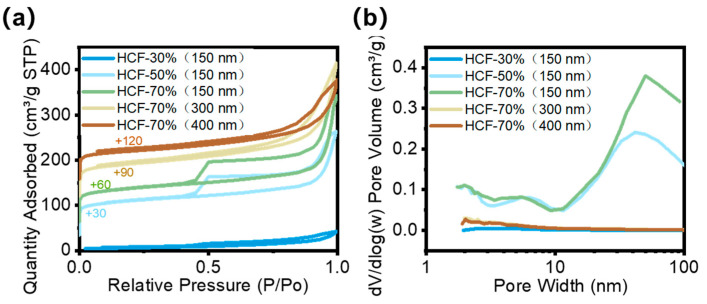
(**a**) The N_2_ adsorption and desorption isotherms of five HCFs calculated by the BET model, (**b**) BJH pore size distribution curves of five HCFs.

The N_2_ adsorption–desorption measurement was utilized to evaluate changes in HCF’s interior porous structure, of which the results are shown in Figure 10 using the BET model. All five HCF groups exhibited hysteresis loops, which could be recognized as type IV isotherms (according to the IUPAC adsorption isotherm classification) [30,36,43], confirming the existence of porous structures. The hysteresis loops of HCF-50% (150 nm) and HCF-70% (150 nm) fibers exhibit saturated adsorption platforms, belonging to the H2 type hysteresis loop in the IUPAC classification, indicating the presence of three-dimensional mesopores (2–50 nm) within them. The hysteresis loops of HCF-70% (300 nm) and HCF-70% (400 nm) belong to the H3 type, while the hysteresis loop of HCF-30% (150 nm) belongs to the H4 type. Neither of them has a clear saturated adsorption platform, indicating an irregular pore structure. The pore size distribution curve calculated by the BJH method (Figure 10b) also confirms the results of the BET model analysis. The internal pores of HCF-50% (150 nm) and HCF-70% (150 nm) fibers are hierarchical porous structures containing mesopores, while the other three groups of HCFs have no obvious peak within a pore size of 100 nm, and may have microporous (<50 nm) and irregular macroscopic porous (>50 nm) structures.

### 3.3. Preparation and Characterization of PCFs

**Figure 11:**
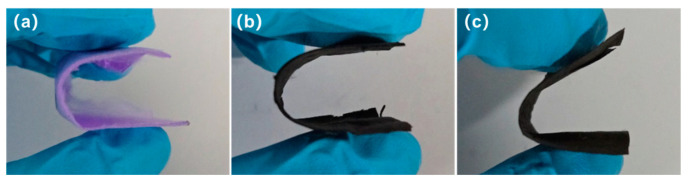
Photographs of (**a**) PAN/ZIF-67 composite fibers; (**b**) HCFs; (**c**) HCF/LA PCFs under bending condition.

In this experiment, LA was utilized as a model small molecule PCM to create phase change composite fibers HCF/LA, and the effect of different HCF porous architectures on PCM’s loading and encapsulation was examined. Photographs of PAN/ZIF-67 composite fibers, HCFs, and HCF/LA PCFs under the bending condition (Figure 11) show that the fibers still maintain good flexibility after loading LA and bending will not cause them to break.

**Figure 12:**
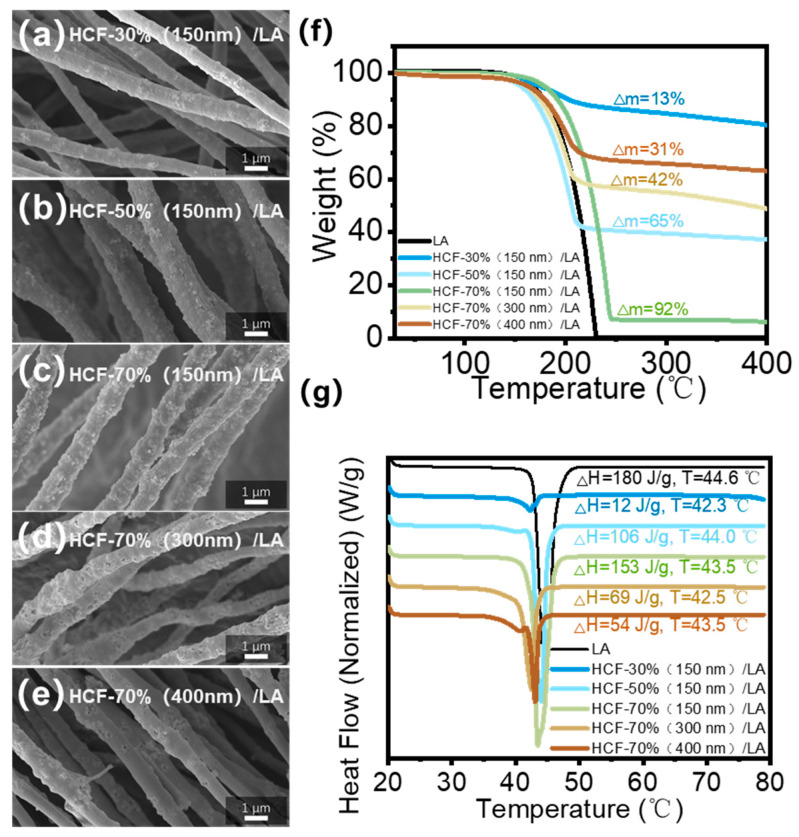
SEM images of (**a**–**e**) five groups of HCF/LA; (**f**) TGA diagram of five groups of HCF loaded with LA; (**g**) DSC images of five groups of HCFs loaded with LA.

Before and after loading with PCMs, the morphological changes of the five groups of HCF/LA phase change composite fibers were observed using SEM. When compared to the original HCFs, the fiber diameter of HCF/LA loaded with LA did not significantly alter, as seen in Figure 12a–e. The loading impact on LA is improved by the HCF-50% (150 nm) and HCF-70% (150 nm) fibers with a hierarchical porous structure primarily made up of mesopores, and the fibers’ pores are filled. The HCF-70% (300 nm) and HCF-70% (400 nm) fibers, on the other hand, have many empty pores on their surface and poor encapsulation effects on PCMs due to their uneven pore topologies. The pore structure, LA loading content, and phase change enthalpy of these prepared HCFs/LAs are summarized in Table 1.

The phase transition process of the HCF/LA fibers and the loading contents of five groups of HCF on LA were examined using TGA and DSC. After washing, the HCF-50% (150 nm) and HCF-70% (150 nm) fibers maintain high loading contents and phase change enthalpy, with loading contents of 65% and 92% (Figure 12f) and phase change enthalpies of 106 J/g and 153 J/g, respectively, demonstrating superior encapsulation effects on PCMs (Figure 12g). Among them, HCF-70% (150 nm) has a loading efficiency of over 90% for PCMs and phase change enthalpies of over 150 J/g, which is superior to most reported phase change composite materials prepared by porous or hollow fibers [22,44,45,46,47], such as the enthalpy value of the phase change fiber (P-HFF) prepared by Wang’s team which was 94.69 J/g when the PEG loading was 65% [44], or the composites of the dual-aligned scaffold and PEG (D-AS/PEG) prepared by Cui’s team with an enthalpy of 146.85 J/g and storage capacity of 83.79% [46].

**Figure 13:**
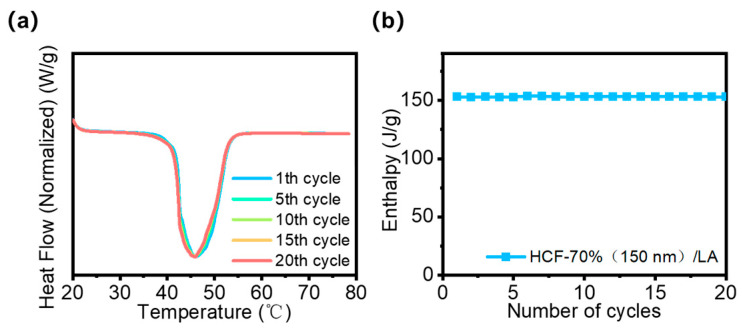
(**a**) DSC thermograms and (**b**) enthalpy change curve of HCF-70% (150 nm)/LA during 20 continuous heating–cooling cycles.

The stability of HCF/LA fibers following several cycles of phase transition is the primary focus. The capacity of a material to retain its phase change characteristics (such as phase change temperature and enthalpy) following several heating and cooling procedures is known as phase change cycle stability. Since PCMs must retain their characteristics after extended use, this is essential for their practical application. It is evident from the data in Figure 13a that the HCF/LA fiber’s phase transition enthalpy hardly changes after 20 cycles, staying at its starting value of 153 J g^−1^. This finding demonstrates the exceptional cycling stability of HCF/LA fibers, which is superior to most reported phase change composite materials prepared by porous or hollow fibers, such as the D-AS/PEG composites which approached about 94% of the original samples and phase change composites with a leakage rate less than 1.7 wt% [46,48], which is highly advantageous for their uses in thermal energy storage and temperature control, among other areas. Furthermore, our finding demonstrates that HCFs may successfully encapsulate LA to create a stable composite material. The HCF/LA fibers did not exhibit LA leakage over several cycles, indicating that the composite’s structural integrity was adequately preserved.

## 4. Conclusions

In this study, electrospinning was used to construct the precursor for the PAN/ZIF-67 composite fibers, and impregnation with a small molecule LA solution produced flexible HCF/LA PCFs with a high energy density. The effects of ZIF-67 doping quantity, fiber orientation, pre-oxidation treatment, and particle size on HCF pore structure and shape were investigated, as was the effect of HCF pore structure on PCM encapsulation. It was found that the oriented fiber structure serves to reduce the likelihood of fracture and breakage of precursor fibers after carbonization, and gradient pre-oxidation can maintain the original fiber morphology of the fibers after carbonization. Fibers may shatter at high carbonization temperatures. A temperature of 800 °C is optimal for maintaining fiber shape. Increased doping contents of 150 nm ZIF-67 can result in better specific surface area and a richer three-dimensional pore structure of the generated porous carbon fibers, but increased particle size of doped ZIF-67 can cause uneven distribution of the porous structure of HCFs. When 150 nm ZIF-67 is doped at 70 wt%, HCFs with high specific surface area and a hierarchical porous structure dominated by mesopores have a higher loading effect on PCMs. Based on the above research, we successfully developed a form of flexible HCFs suitable for the encapsulation of PCMs, with a high loading capacity for LA (92%) and a phase change enthalpy value of 153 J g^−1^. The heating–cooling cycle experiments have demonstrated the cyclic stability of HCF/LA for latent heat storage. HCF/LA has excellent thermal regulation performance and high flexibility, and has great application potential in wearable thermal management, intelligent temperature regulation, and hyperthermia.

## Figures and Tables

**Table 1:** Porous structure, LA loading content, and phase change enthalpy of prepared HCF/LA.

Name	Porous Structure	LA Loading Content (wt%)	Phase Change Enthalpy (J/g)
HCF-30% (150 nm)/LA	Microporous	13	12
HCF-50% (150 nm)/LA	Mesoporous	65	106
HCF-70% (150 nm)/LA	Mesoporous	92	153
HCF-70% (300 nm)/LA	Macroporous	42	69
HCF-70% (400 nm)/LA	Macroporous	31	54

## Data Availability

Data are contained within the article.

## Abbreviations

Name	Abbreviations
Phase change materials	PCMs
Phase change fibers	PCFs
Hollow carbon fibers	HCFs
Lauric acid	LA
Metal-organic frameworks	MOFs
N. N-dimethylformamide	DMF
Cobalt nitrate hexahydrate	Co(NO_3_)_2_·6H_2_O
2-methylimidazole	2-MI
Scanning electron microscopy	SEM
Transmission electron microscopy	TEM
X-ray diffraction	XRD
Brunner–Emmet–Teller measurements	BET
Barrett–Joerner–Halenda model	BJH
Differential scanning calorimetry	DSC
Thermo-gravimetric analyzer	TGA
